# Determining Ligand and Ion-Induced Conformational Changes in Serotonin Transporter with Its Fluorescent Substrates

**DOI:** 10.3390/ijms231810919

**Published:** 2022-09-18

**Authors:** Mu Li, Qingyang Chen, Yuan-Wei Zhang

**Affiliations:** School of Life Sciences, Guangzhou University, Guangzhou 510006, China

**Keywords:** serotonin transporter, conformational mechanism, antidepressants, fluorescent substrates, APP^+^, ASP^+^, confocal imaging analysis

## Abstract

Conformational changes are fundamental events in the transport mechanism. The serotonin transporter (SERT) catalyzes reuptake of the neurotransmitter serotonin after its release by serotonergic neurons and is the molecular target for antidepressant drugs and psychostimulants. Despite significant progress in characterizing the structure–function relationship of SERT, its conformational mechanism has not been fully understood. We present here a cell-based method for determining conformational changes in SERT with its fluorescent substrates by fluorescence imaging analysis. This method fluorometrically measures accessibility of strategically positioned cysteine residues in the substrate permeation pathway to calculate the rate constants of reactivity with MTS reagents in live or permeabilized cells. We validated this method by investigating ligand and ion-induced conformational changes in both the extracellular and cytoplasmic pathways of SERT. Furthermore, we applied this method for examining the influence of Cl^−^ binding and vilazodone inhibition on SERT conformation. Our results showed that Cl^−^ ion, in the presence of Na^+^, facilitates the conformational conversion from outward to inward open states, and that vilazodone binding stabilizes SERT in an outward open and inward-closed conformation. The present work provided insights into the conformational mechanism of SERT and also indicated that the cell-based fluorometric method is robust, straightforward to perform, and potentially applicable to any monoamine transporters in exploring the transport mechanism and mechanism of action of therapeutic agents for the treatment of several psychiatric disorders.

## 1. Introduction

The serotonin transporter (SERT) is a presynaptic plasma membrane protein responsible for the reuptake of serotonin (5-hydroxytrypatamine, 5-HT) after its release by serotonergic neurons [1,2]. It belongs to a large family of neurotransmitter sodium symporters (NSS), which also includes transporters for dopamine (DAT), norepinephrine (NET), γ-aminobutyric acid (GAT), and glycine (GlyT). Together with DAT and NET, SERT is also a member of a subgroup transporters in the NSS family that symport monoamines with Na^+^ and Cl^−^ ions. Among these transporters, SERT is of particular interest in neuropharmacology because it is the major target of drugs of abuse, such as cocaine and amphetamines, and antidepressants, such as fluoxetine, citalopram, and imipramine [3,4,5,6,7].

The NSS transporters are thought to transport their substrates by an alternating access mechanism in which the central binding site is alternately exposed to the extracellular or cytoplasmic medium for substrate binding and release by conformational changes that open and close the substrate permeation pathway [8,9,10,11]. As conformational changes undergo to transport substrates across the membrane, it becomes essential to understand how substrates, ions, and other ligands exert their effects on conformational changes [8,12,13,14,15,16,17].

The crystal structures at several conformational states of leucine transporter (LeuT), a homolog of the NSS family, provide insight into conformational changes during transport [18,19,20]. In addition, the recently resolved structures of DAT, SERT, and GlyT1 reveal these transport proteins hold a common LeuT fold, indicating that they share a similar conformational mechanism [21,22,23,24,25,26]. However, these crystallographic studies have not fully addressed how the conformational changes are regulated to couple substrate and ion movements by their binding events. Biochemical and molecular dynamics simulation analyses for LeuT have shown that Na^+^ binding stabilizes the transporter in an outward open conformation, and that the subsequent binding of substrate overcomes the effect of Na^+^, allowing conformational conversion and substrate release intracellularly [14,27,28,29,30]. For SERT, resonance energy transfer measurements with a double-tagged construct, in which a fluorescence donor and acceptor were attached to the N and C-terminus, respectively, were used for examining intramolecular distance changes between the two termini in response to ligand or ion binding and substrate transport [31,32]. Because the NSS transporters are known to form oligomers [33], intermolecular fluorescence resonance energy transfer signal might obscure ion or ligand-induced conformational changes within the transporter molecule [31]. In addition, it is also doubtful whether ion, substrate, or ligand-induced conformational rearrangements occurred in the core region of the transporter protein can be exactly reflected by movements of the unessential moieties for SERT catalytic function, such as the N or C-terminal tail.

We previously developed an assay based on accessibility of reactive cysteine residues in the substrate permeation pathway to measure conformational changes in response to substrate, ion, or ligand binding in SERT and other members in the NSS family [8,15,16,34,35]. This assay has been recently applied for the study of ion-substrate coupling mechanism with LeuT and GlyT1 [36,37]. In these analyses, radiolabeled substrates or ligands were generally employed to monitor the inhibition of substrate transport or ligand binding by chemical modification of the transporter proteins with cysteine reagents. However, high cost, instability, short half-life, and high-standard requirements for environment and safety limit the use of radioactive reagents. Moreover, since large populations of cells are sampled in the radioactive assays, differences in protein expression and localization may mask effects limited to a specific cell population [38].

The fluorescent substrates of monoamine transporters, 4-[4-(dimethylamino) phenyl]-1-methylpyridinium (APP^+^) and 4-(4-(dimethylamino) styryl)-N-methylpyridinium (ASP^+^), have emerged as a powerful tool to fluorometrically investigate the substrate binding stoichiometry [39], protein kinase-mediated regulation [38], K^+^ requirements [40] of monoamine transporters, and the mechanism of action of novel antidepressants [41]. We present here a cell-based method for using these fluorescent substrates to assess conformational changes in response to ligand and ion binding in SERT. We performed fluorescence imaging analyses to measure ligand and ion-induced accessibility changes in both the extracellular and cytoplasmic pathways, which were reflected by the effects of chemical modification on transport or binding activity of SERT. Furthermore, we applied this fluorometric method for investigating the influence of Cl^−^ binding and vilazodone inhibition on SERT conformation. Our results showed that the method is straightforward to perform, robust, and potentially applicable to any monoamine transporters in the NSS family.

## 2. Results

### 2.1. Transport of APP^+^ or ASP^+^ by SERT Is Fluoxetine-Sensitive and Requires Both Na^+^ and Cl^−^

**Figure 1:**
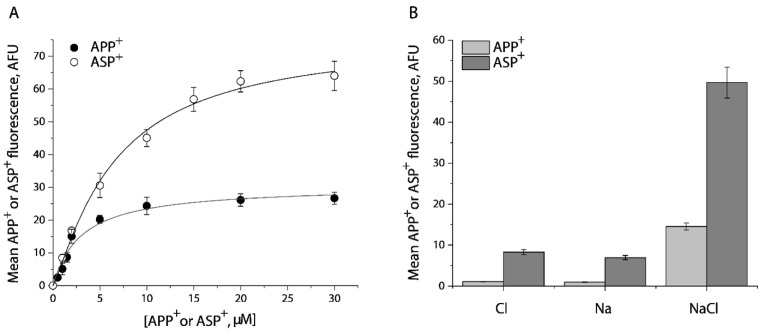
Kinetic analysis and ion requirements for APP^+^ or ASP^+^ uptake by SERT. (**A**) Kinetic analysis for APP^+^ or ASP^+^ uptake by SERT. The cells stably expressing WT-SERT were incubated with APP^+^ or ASP^+^ over a range of concentrations, as indicated, in the absence or presence of 10 μM fluoxetine at 22 °C for 5 min. After washing to remove excess APP^+^ or ASP^+^, the accumulated fluorescence within the cells (*n* ≥ 30) were counted and normalized to the cell areas, as described under “Materials and Methods”. Nonspecific transport for APP^+^ or ASP^+^ was measured in the presence of 10 μM fluoxetine and was subtracted to give APP^+^ or ASP^+^ transport, respectively. The graph shows a representative experiment with APP^+^ or ASP^+^ influx expressed as mean fluorescence (AFU). The experiment was repeated twice with similar results. The *K_m_* value for APP^+^ or ASP^+^ was 2.63 ± 0.06 μM or 8.52 ± 0.15 μM, and *V_max_* for APP^+^ or ASP^+^ was 30.5 ± 1.4 AFU, or 75.8 ± 5.3 AFU, respectively. These *K_m_* and *V_max_* values represent mean ± SEM of three experiments. (**B**) Ion-dependence of APP^+^ or ASP^+^ uptake. APP^+^ or ASP^+^ uptake was measured by incubating the cells stably expressing WT- SERT with APP^+^ at 2 μM or ASP^+^ at 10 μM in HEPES buffer containing 150 mM NMDGCl, sodium isethionate, or NaCl, respectively. Error bars represent SEM of three experiments.

To determine the specificity of APP^+^ or ASP^+^ transport by SERT, we performed kinetic analysis for the fluorescent substrates in the presence or absence of fluoxetine. As shown in Figure 1A, APP^+^ is a better substrate than ASP^+^. The *K_m_* value for APP^+^ or ASP^+^ was 2.63 ± 0.06 μM or 8.52 ± 0.15 μM, respectively. At the highest substrate concentration used, approximately 95% of APP^+^ or 84% of ASP^+^ accumulation was blocked by preincubation with 10 μM fluoxetine, respectively. The residual fluorescence was considered to be nonspecific uptake by the cells. It is notable that ASP^+^ showed a higher fluorescent signal than APP^+^.

Figure 1B shows ion requirements for transport of APP^+^ or ASP^+^ by SERT. Consistent with SERT transport of its endogenous substrate, 5-HT, Na^+^, or Cl^−^ alone did not support transport of either APP^+^ or ASP^+^. SERT requires the coexistence of Na^+^ and Cl^−^ to trigger its transport ability for APP^+^ or ASP^+^.

### 2.2. Cysteine Mutants in the Substrate Permeation Pathway Show Comparable Kinetic Parameters to the WT-SERT

**Figure 2:**
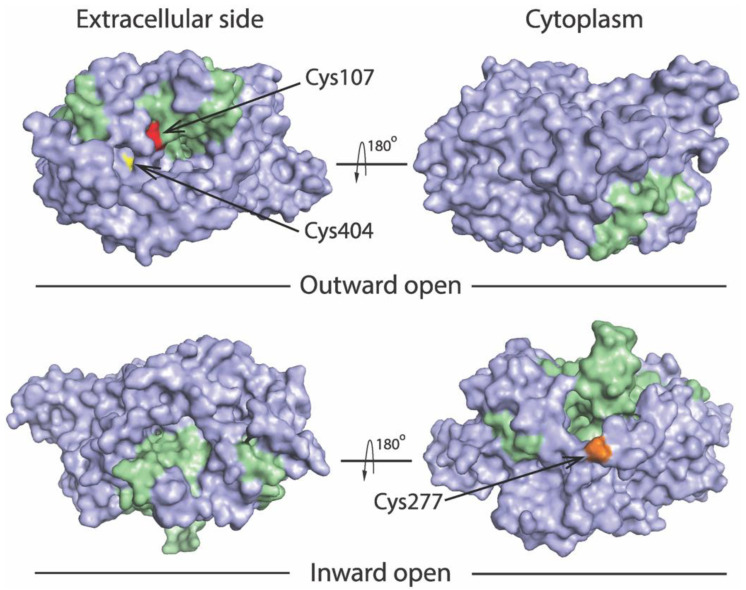
Location of the cysteine residues used to probe pathway accessibility. Views of the outward open (PDB ID code 7LIA) and the inward open (PDB ID code 7LI9) SERT structures [25] are shown from extracellular side (**left**) and cytoplasm (**right**). The bundle domain (TMs 1, 2, 6, and 7) and other part of the protein are colored green and gray, respectively. The modified residues Y107C (red), S404C (yellow), and S277C (gold) are shown to illustrate accessibility of Cys107 and Cys404 to the extracellular pathway, and Cys277 to the cytoplasmic pathway.

We previously utilized an assay based on accessibility of reactive cysteine residues in the extracellular or cytoplasmic substrate permeation pathway, to measure conformational changes in SERT and other members in the NSS transporter family [8,14,15,34,36,37]. The corresponding cysteine mutants selected for this study include Y107C/C109A and S404C/C109A in the extracellular pathway and S277C/X5C in the cytoplasmic pathway (Figure 2). Both Cys107 and Cys404 are exposed to the extracellular medium in the outward open conformation, whereas they are buried in the inward open conformation. By comparison, Cys277 is buried in the outward open conformation, but exposed to cytoplasmic medium in the inward open conformation.

The two cysteine mutants in the extracellular pathway were constructed in C109A background, whereas the cytoplasmic mutant was in X5C background, which lacks accessible cysteine residues in the membrane preparations [15,42,43,44,45,46]. To these mutants, we performed kinetic analysis for transport of the fluorescent substrates. The *K_m_* values for APP^+^ and ASP^+^ in these cysteine mutants were comparable to those in the WT (Table 1). The *V_max_* values of these mutants for APP^+^ were in a range of 26-39 mean arbitrary fluorescence units (AFU), which are not statistically different from that of the WT. On the other hand, when ASP^+^ was used as a substrate, Y107C/C109A showed a *V_max_* similar to that of the WT, S404C/C109A or S277C/X5C, however, had a *V_max_* value of approximate 70% or 50% of the WT, respectively.

### 2.3. MTSET Modification of the Cysteine Residues in the Extracellular Pathway Inhibits APP^+^ Uptake

**Figure 3:**
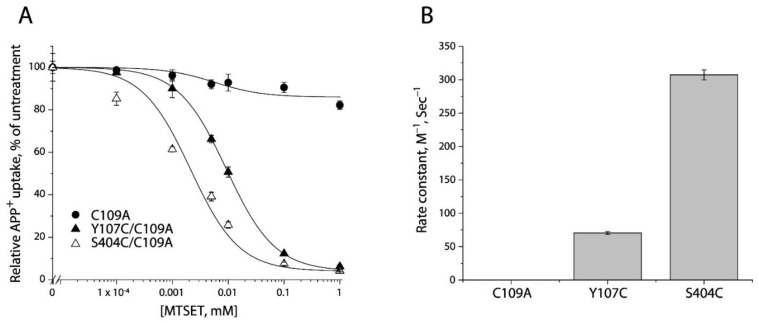
Reactivity of the extracellular cysteine mutants with MTSET. (**A**) MTSET concentration-dependent inhibition of APP^+^ uptake. The cells stably expressing C109A, Y107C/C109C, or S404C/C109A were incubated for 15 min with indicated concentrations of MTSET. The MTSET concentration giving half-maximal inhibition of APP^+^ accumulation in the cells was used to calculate inactivation rate constants as plotted in (**B**). In this experiment, half-maximal MTSET concentrations were >1000 mM for C109A, 0.011 ± 0.006 mM for Y107C/C109A, and 0.0025 ± 0.0008 mM for S404C/C109A, respectively. Representative images of APP^+^ uptake by C109A, Y107C/C109A, or S404C/C109A under various MTSET concentrations were shown in Supplemental Figure S1. The experiment was repeated twice with similar results. (**B**) Rate constants of MTSET inhibition of APP^+^ uptake. At the half-maximal concentration, the half-time is the assay time (15 min) and the first-order rate constant is 7.7 × 10^−4^ s^−1^. This rate constant was divided by the half maximal MTSET concentration from (**A**) to obtain the pseudo first-order modification rate constant. Error bars represent SEM of three experiments.

2-(Trimethylammonium) ethyl methanethiosulfonate bromide (MTSET), a membrane impermeant reagent, was employed to examine accessibility of cysteine residues in the extracellular pathway, based on its effect on APP^+^ uptake into the cells. As shown in Figure 3A and Supplemental Figure S1 (representative fluorescence images), MTSET had little effect on transport activity of the background construct, C109A. In contrast, MTSET modification dramatically inhibited APP^+^ uptake by Y107C/C109A or S404C/C109A in a concentration-dependent manner. The MTSET concentration leading to half-maximal inhibition, reflecting the extent of modification over a range of MTSET concentrations (0.1 μM to 1 mM), was determined and converted to a pseudo first-order rate constant (Figure 3B). Depending on their positions in the extracellular pathway, S404C/C109A reacted with the aqueous MTSET faster than Y107C/C109A by 4-5-fold.

### 2.4. Determining Ligand-Induced Conformational Changes in the Extracellular Pathway by MTSET Inhibition of APP^+^ Uptake in Live Cells

**Figure 4:**
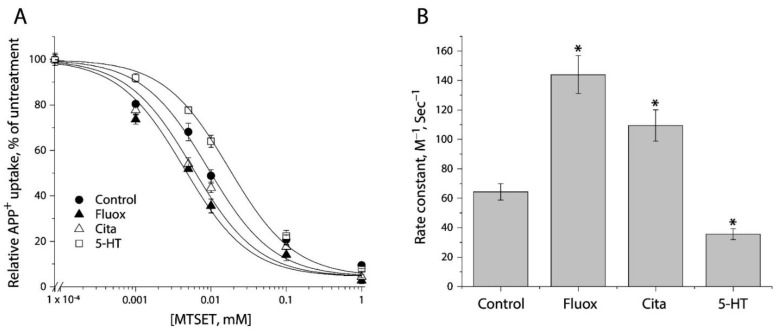
The effects of antidepressants and substrate on MTSET inactivation of APP^+^ uptake by Y107C/C109A. (**A**) MTSET concentration-dependent inhibition of APP^+^ uptake. The cells stably expressing Y107C/C109A were treated for 15 min with indicated concentrations of MTSET in the absence or presence of 10 μM fluoxetine (Fluox), citalopram (Cita) or 5-HT. After washing to remove excess MTSET and ligands, APP^+^ uptake was measured as described under “Materials and Methods”. The graph shows a representative experiment. The experiment was repeated twice with similar results. (**B**) Rate constants of MTSET inhibition of APP^+^ uptake. The IC_50_ values of MTSET inhibition were used to calculate rate constants as described under “Materials and Methods”. Error bars represent SEM from three experiments. Asterisks indicate statistically significant changes (*p* < 0.05) in rate constant compared to the control (MTSET alone).

We examined the effects of antidepressants (fluoxetine and citalopram) and substrate (5-HT) on MTSET modification of Y107C/C109A mutant. Following exposure to these ligands for 5 min, the cells stably expressing Y107C/C109A were incubated with MTSET over a range of concentrations (1 μM to 1 mM) for an additional 15 min. At the end of this incubation, cells were washed free of MTSET and ligands into KRH buffer containing NaCl and APP^+^. Figure 4A shows MTSET concentration-dependent inhibition of APP^+^ uptake under various ligand treatments. Treatment with either fluoxetine or citalopram resulted in a shifting of the inhibition curve against APP^+^ uptake to the lower MTSET concentration range compared to the control, indicating MTSET reactivity became faster than the control. In contrast, incubation with substrate, 5-HT, in the presence of both Na^+^ and Cl^−^ ions induced the inhibition curve shifting to the higher MTSET concentration range, reflecting MTSET reactivity was slower than the control.

Figure 4B shows rate constants of MTSET reactivity, which were determined by the IC_50_ values of APP^+^ uptake inhibition (Figure 4A). In comparison with the control rate constant, both fluoxetine and citalopram treatments increased MTEST reactivity by 1.7–2.2-fold, indicating that binding of these antidepressant drugs increased accessibility of Y107C/C109A to the aqueous MTSET. On the other hand, 5-HT decreased the rate constant by approximate 2 folds.

**Figure 5:**
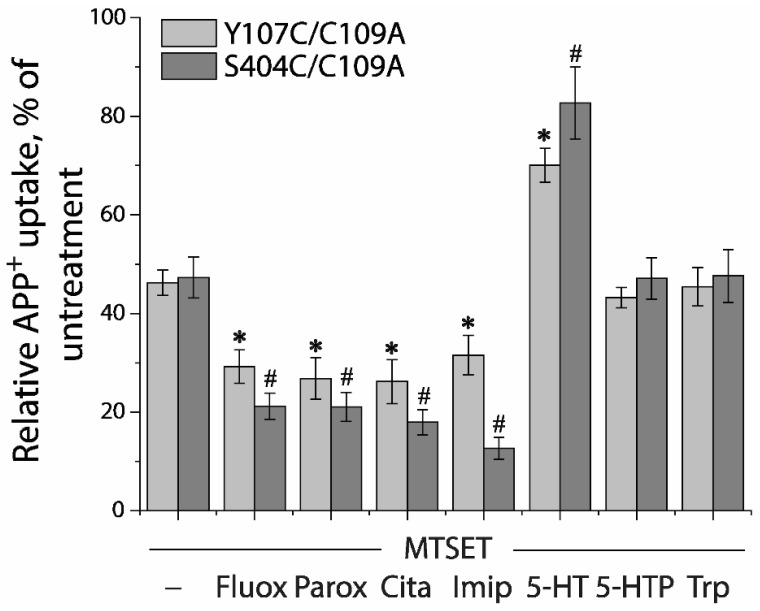
Ligand-induced accessibility changes in the extracellular pathway. The cells stably expressing Y107C/C109A or S404C/C109A were preincubated with indicated ligands and MTSET at a concentration of 0.01 mM for Y107C/C109A or 0.002 mM for S404C/C109A at 22 °C for 15 min, respectively. At the end of this incubation, cells were washed free of MTSET and ligands into KRH buffer containing NaCl and APP^+^. APP^+^ uptake was measured as described under “Materials and Methods”. Error bars represent SEM from three experiments. Asterisks and hashtags indicate statistically significant changes (*p* < 0.05) in the accumulated APP^+^ fluorescence compared with the corresponding control (MTSET alone). Fluox, fluoxetine; Paro, paroxetine; Cita, citalopram; Imip, imipramine; 5-HTP, 5-hydroxytryptophan; Trp, L-tryptophan.

Next, we utilized a simplified assay to assess ligand-induced conformational changes in the extracellular pathway with both Y107C/C109A and S404C/C109A mutants (Figure 5). In this assay, MTSET was added at a constant concentration of the IC_50_ value (10 μM for Y107C/C109A or 2 μM for S404C/C109A) into the KRH buffer in the presence of indicated ligands. Altered ligand addition was present only during the incubation with MTSET and not during the APP^+^ transport measurements. We measured the ability of ligands to influence the reactivity of Y107C/C109A or S404C/C109A with MTSET. Antidepressant drugs, such as fluoxetine, paroxetine, citalopram, and imipramine, which stabilize the outward open conformation of SERT [16,17,23,47,48], markedly increased the reactivity of Y107C/C109A or S404C/C109A, promoting MTSET inactivation of the mutants (Figure 5). In contrast, substrate 5-HT significantly decreased the reactivity of Y107C/C109A or S404C/C109A by protecting the mutants from inactivation, consistent with our previous observation that 5-HT induced conformational conversion from outward open to inward open [9,15]. On the other hand, precursor compounds for 5-HT biosynthesis, 5-hydroxytryptophan (5-HTP), and tryptophan (Trp) had little effect on the reactivity of either Y107C/C109A or S404C/C109A (Figure 5).

### 2.5. Measuring Conformational Changes in the Cytoplasmic Pathway with ASP^+^

**Figure 6:**
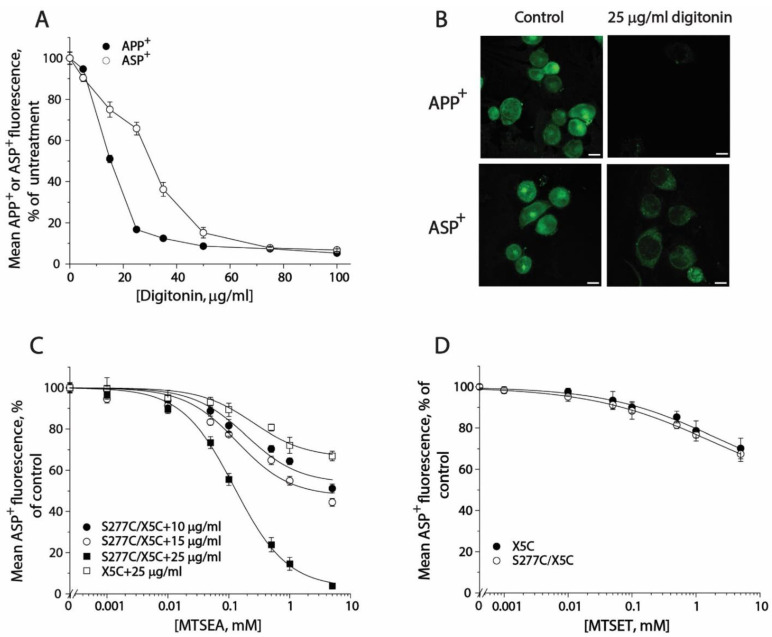
The effects of digitonin on APP^+^ or ASP^+^ accumulation and accessibility of S277C/X5C to MTS reagents. (**A**) The effects of digitonin on APP^+^ and ASP^+^ accumulation. The cells stably expressing WT-SERT were incubated with 2 μM APP^+^ or 10 μM ASP^+^ in the absence or presence of digitonin over a range of concentrations (10–100 μg/mL) at 22 °C for 5 min. After 3 × rapid washing with KRH buffer, APP^+^ or ASP^+^ accumulation was measured as described under “Materials and Methods”. The graph shows a representative experiment. The experiment was repeated twice with similar results. (**B**) Images of APP^+^ or ASP^+^ fluorescence (green) accumulated in the presence or absence of 25 μg/mL digitonin (scale bar: 10 μm). These representative images were parallelly acquired from one experiment. The experiment was repeated twice with similar results. (**C**) MTSEA concentration-dependent inhibition of ASP^+^ binding of S277C/X5C. The cells stably expressing S277C/X5C were incubated with MTSEA over a range of concentrations (1 μM to 5 mM) in the absence or presence of 10, 15, or 25 μg/mL digitonin at 22 °C for 5 min. After 3 × washing, ASP^+^ binding to the cell membrane was measured as described under “Materials and Methods”. The effect of MTSEA on ASP^+^ binding of the background construct, X5C, in the presence of 25 μg/mL digitonin was also parallelly examined. The graph shows a representative experiment. Two more experiments were performed with similar results. (**D**) The effects of MTSET on ASP^+^ binding of S277C/X5C and X5C. The cells stably expressing S277C/X5C or X5C were incubated with 10 μM ASP^+^ with MTSET over a range of concentrations (1 μM to 5 mM) in the absence or presence of 25 μg/mL digitonin for 5 min. After 3 × rapid washing, ASP^+^ binding was measured as described under “Materials and Methods”. The graph shows a representative experiment. Two more experiments were performed with similar results.

In order to monitor conformational changes in the cytoplasmic pathway, we developed a cell-based method for accessibility measurements of a strategically placed cysteine residue, S277C, as an indicator using fluorescence imaging analysis. Although it has been reported that 2-aminoethyl methanethiosulfonate hydrobromide (MTSEA) is more membrane permeable than other MTS reagents, we did not observe MTSEA modification of the cysteine residue in the cytoplasmic pathway with intact cells during 15-min incubation. In this study, we employed digitonin to permeabilize the cell membrane and examined its effects on substrate uptake and binding. As shown in Figure 6A, digitonin treatment dramatically decreased APP^+^ accumulation within the cells. In contrast, ASP^+^ fluorescence was slightly decreased when digitonin was used at low concentrations. There was more than 60% ASP^+^ fluorescence retained in the cells when 25 μg/mL digitonin was added. Figure 6B shows APP^+^ and ASP^+^ fluorescence images in the cells with or without 25 μg/mL digitonin addition. In comparison with control (upper left), there was only a trace of APP^+^ fluorescence detected in the cells (Figure 6B, upper right), indicating SERT lost its ability to uptake APP^+^ under 25 μg/mL digitonin treatment. On the other hand, compared with control (Figure 6B, bottom left), more than 60% of ASP^+^ fluorescence was retained under the same treatment (Figure 6B, bottom right). It is notable that digitonin addition led to a loss of ASP^+^ intracellular accumulation by SERT; the ASP^+^ fluorescence, however, was detected exclusively in the cell membrane (Figure 6B, bottom right), suggesting that the cell surface SERT was functional to bind ASP^+^ in the presence of 25 μg/mL digitonin.

Then, we examined the effect of digitonin concentration on MTSEA modification of S277C/X5C and X5C background construct (Figure 6C). When digitonin was added at a concentration of 10 or 15 μg/mL, MTSEA at the highest concentration used only inhibited ASP^+^ binding by 40% or 50%, respectively. By comparison, addition of 25 μg/mL digitonin was effective to lead to a full inhibition of ASP^+^ binding by MTSEA. It is noted that MTSEA treatment also resulted in a partial inhibition of ASP^+^ binding with X5C when 25 μg/mL digitonin was used (Figure 6C). However, the rate constant for MTSEA reactivity with S277C/X5C is approximate 250-fold higher than that for its X5C background (11.97 M^−1^, S^−1^ vs. 0.05 M^−1^, S^−1^). Hence, it is reasonable to ignore the effect of digitonin on the X5C background. Furthermore, we examined the effect of digitonin on the MTSET reactivity with S277C/X5C. As shown in Figure 6D, Cys277 was not accessible to MTSET even when 25 μg/mL digitonin was used. Taken together, our results indicate that it is feasible to measure accessibility of Cys277 in the cytoplasmic pathway in a cell-based fluorometric assay, in which 25 μg/mL digitonin is effective to permeabilize the cell membrane, allowing MTSEA fully access to the cysteine residue.

### 2.6. Evaluating Ligand-Induced Conformational Changes in the Cytoplasmic Pathway by MTSEA Inhibition of ASP^+^ Binding

**Figure 7:**
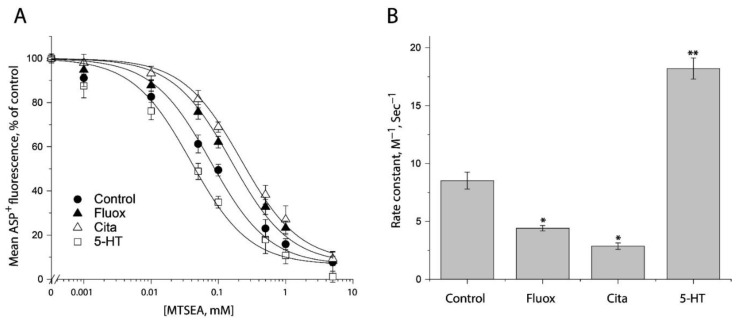
Ligand-induced accessibility changes in the cytoplasmic pathway. (**A**) The effects of ligands on MTSEA inhibition of ASP^+^ binding of S277C/X5C. The cells stably expressing S277C/X5C were treated for 5 min with 25 μg/mL digitonin and indicated concentrations of MTSEA in the absence or presence of 10 μM fluoxetine (Fluox), citalopram (Cita) or 5-HT. After washing to remove excess MTSEA and ligands, ASP^+^ binding was measured as described under “Materials and Methods”. The graph shows a representative experiment. The experiment was repeated twice with similar results. (**B**) Rate constants of ligand-induced MTSEA inhibition of ASP^+^ binding of S277C/X5C. The IC_50_ values of MTSEA inhibition of ASP^+^ binding were converted to rate constants. Error bars represent SEM from three experiments. * *p* < 0.05; ** *p* < 0.01.

In an attempt to examine ligand-induced conformational changes in the cytoplasmic pathway, in the presence of 25 μg/mL digitonin, we treated the cells stably expressing S277C/X5C with MTSEA over a range of concentrations (1 μM to 5 mM) under indicated ligand treatment. After washing free of ligand and MTSEA into KHR buffer containing NaCl and ASP^+^, we measured MTSEA inhibition of ASP^+^ binding. Figure 7A shows the inhibition under antidepressant and substrate treatments. In comparison with control (without ligand addition), antidepressants decreased but substrate increased MTSEA inhibition of ASP^+^ binding, reflecting their different effects on accessibility of S277C/X5C. Figure 7B shows the rate constants of MTSEA modification of S277C/X5C. Fluoxetine or citalopram significantly decreased the rate constant by ~50% or ~67%, respectively, indicating that antidepressant binding stabilizes an inward-closed conformation. In contrast, in the presence of NaCl, the substrate, 5-HT, increased the rate constant by ~2 folds, reflecting 5-HT together with both Na^+^ and Cl^−^ stabilizes an inward open conformation.

### 2.7. Application of the Fluorometric Assay for Studying Conformational Mechanism of Transport and Antidepressant Action

The mechanism of the NSS transporter action involves conformational changes that alternately open and close aqueous permeation pathways for substrate binding from extracellular medium and release to cytoplasm. According to results with LeuT, Na^+^ binding to the Na2 site stabilizes a closed cytoplasmic pathway, allowing the extracellular pathway to open for substrate binding [14,49]. The subsequent substrate binding stabilizes the interactions between the scaffold and bundle domains that overcome the effect of Na^+^ at the Na2 site and act to stabilize the closed extracellular pathway, allowing the cytoplasmic pathway to open and substrates to dissociate [36,50]. Most of the NSS transporters require Cl^−^ for their function, while the prokaryotic LeuT does not [36]. The conformational role of Cl^−^ in SERT, however, has not been revealed yet.

**Figure 8:**
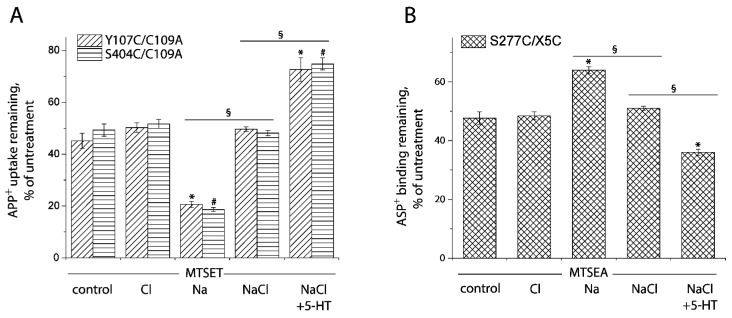
Ion-induced accessibility changes in both the extracellular and cytoplasmic pathways. (**A**) Ion-induced accessibility changes in the extracellular pathway. The cells stably expressing Y107C/C109A or S404C/C109A were preincubated with MTSET at 0.01 mM for Y107C/C109A or 0.002 mM for S404C/C109A in the presence of 150 mM NMDG gluconate, NMDGCl, sodium isethionate, NaCl, or NaCl + 5-HT (10 μM) for 15 min, respectively. After 3 × rapid washing, APP^+^ uptake was measured as described under “Materials and Methods”. Error bars represent SEM from three experiments. Asterisks and hashtags indicate statistically significant changes (*p* < 0.05) compared with the corresponding control (NMDG gluconate). The signs *§* indicate significant changes between two conditions as indicated. (**B**) Ion-induced accessibility changes in the cytoplasmic pathway. The cells stably expressing S277C/X5C were treated for 5 min with 25 μg/mL digitonin and 0.12 mM MTSEA in the presence of 150 mM NMDG gluconate, NMDGCl, sodium isethionate, NaCl, or NaCl + 5-HT (10 μM). ASP^+^ binding was measured as described under “Materials and Methods”. Error bars represent SEM from three experiments. Asterisks indicate statistically significant changes (*p* < 0.05) compared with control (NMDG gluconate). The signs § indicate significant changes between two conditions as indicated. Representative images of APP^+^ uptake by Y107C/C109A and S404C/C109A or ASP^+^ binding by S277C/X5C under various ion conditions were shown in Supplemental Figure S2A,B, respectively.

In this study, we applied the cell-based fluorometric method for investigating the effects of Cl^−^ ion on accessibility of the cysteine residues in both the extracellular and cytoplasmic pathways (Figure 8 and Figure S2). In the absence of Na^+^, Cl^−^ had little effect on SERT conformation. Na^+^, however, increased the reactivity of Cys107 or Cys404 and decreased Cys277 reactivity, consistent with previous observation with the corresponding cystine mutants in LeuT (Figure 8A,B). Addition of Cl^−^ and 5-HT reversed the effect of Na^+^ and decreased Cys107 or Cys404 reactivity while it increased Cys277 reactivity, consistent with closing the extracellular pathway and opening the cytoplasmic pathway (rightmost columns in Figure 8A,B). Strikingly, addition of Cl^−^, in the presence of Na^+^, led to small but significant changes in accessibility in both the pathways (Figure 8A,B). These changes were in the opposite direction to the Na^+^-induced stabilization of the outward-open conformation but not as large as the change due to addition of both 5-HT and Cl^−^. These results suggest that, in the presence of Na^+^, Cl^−^ caused a partial closure of the extracellular pathway and a partial opening of the cytoplasmic pathway. Moreover, under these conditions, addition of 5-HT further decreased accessibility in the extracellular pathway and increased it in the cytoplasmic pathway, leading to a conformational conversion from an outward open state to an inward open state.

**Figure 9:**
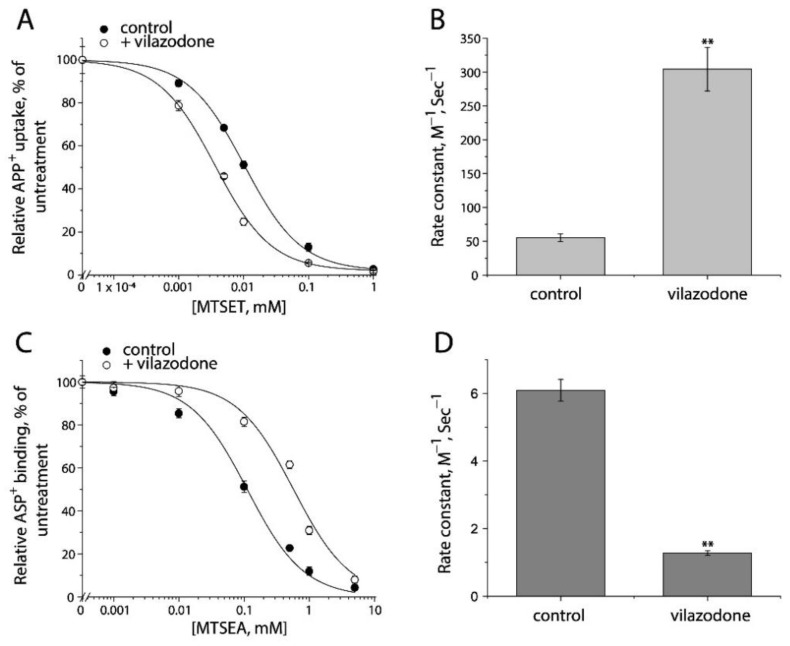
The effects of vilazodone on SERT conformation. (**A**) The effect of vilazodone on MTSET inhibition of APP^+^ uptake by Y107C/C109A. The cells stably expressing Y107C/C109A were treated at 22 °C for 15 min with indicated concentrations of MTSET in the absence or presence of 10 μM vilazodone. After washing to remove excess MTSET and vilazodone, APP^+^ uptake was measured as described under “Materials and Methods”. The graph shows a representative experiment. The experiment was repeated twice with similar results. (**B**) Rate constants of MTSET inhibition of APP^+^ uptake by Y107C/C109A. The IC50 values of MTSET inhibition were used to calculate rate constants as described under “Materials and Method”. Error bars represent SEM from three experiments. Asterisks indicate statistically significant changes in rate constant compared to the control (MTSET alone). ** *p* < 0.01. (**C**) The effects of vilazodone on MTSEA inhibition of ASP^+^ binding by S277C/X5C. The cells stably expressing S277C/X5C were treated for 5 min with 25 μg/mL digitonin and indicated concentrations of MTSEA in the absence or presence of 10 μM vilazodone. After washing to remove excess MTSEA and vilazodone, ASP^+^ binding was measured as described under “Materials and Methods”. The graph shows a representative experiment. The experiment was repeated twice with similar results. (**D**) Rate constants of vilazodone-induced MTSEA inhibition of ASP^+^ binding of S277C/X5C. The IC50 values of MTSEA inhibition of ASP^+^ binding were converted to rate constants. Error bars represent SEM from three experiments. ** *p* < 0.01. Representative images of APP^+^ uptake by Y107C/C109A and ASP^+^ binding by S277C/X5C under various MTSET or MTSEA concentrations were shown in Supplemental Figure S3A,B, respectively.

Furthermore, we examined the conformational mechanism of vilazodone action on SERT by using the cell-based fluorometric assay. Vilazodone is an antidepressant drug with a novel underlying mechanism of action by which it binds to an allosteric site in SERT, and thereby non-competitively inhibits 5-HT transport by SERT [48]. However, the influence of vilazodone on SERT conformation is unknown. We measured vilazodone-induced conformational changes based on accessibility of the cysteine residues in both the extracellular and cytoplasmic pathways in live or digitonin-permeabilized cells (Figure 9 and Figure S3). As shown in Figure 9A,B, vilazodone increased Cys107 reactivity with MTSET in the intact cells stably expressing Y107C/C109A mutant, resulting in a higher rate constant compared to the control. In contrast, vilazodone treatment decreased Cys277 reactivity with MTSEA in the digitonin-permeabilized cells stably expressing S277C/X5C, leading to a lower rate constant than the control (Figure 9C,D). These results support the proposal that vilazodone binding to the allosteric site stabilizes SERT in an outward open and inward-closed conformation, thus inhibits the conformational conversion essential for the transport function.

## 3. Discussion

We report here a new cell-based method for determining conformational changes in SERT by using its fluorescent substrates. This method fluorometrically measures accessibility changes in the cysteine residues in both the extracellular and cytoplasmic substrate permeation pathways in response to ion or ligand binding and substrate transport. By using this method, we investigated the conformational mechanism of Cl^−^ binding and vilazodone inhibition. Our aim in developing this assay was to provide a straightforward to perform, robust, and radioactive reagent-free method to investigate the conformational mechanism for monoamine transporters. With this method, accessibility measurement is performed using fluorescent substrates to monitor the effects of chemical modification of the reactive cysteine residues by MTS reagents on transport or ligand binding activity in live or permeabilized cells by confocal imaging analysis.

The monoamine transporters, SERT, DAT, and NET, all uptake both APP^+^ and ASP^+^ [38,39,51,52,53,54,55,56,57,58]. APP^+^, however, is a better substrate than ASP^+^ for SERT [39], whereas ASP^+^ has been characterized to be an effective fluorescent reporter for uptake activity for both DAT and NET [54,55,56]. Hence, these fluorescent substrates can be used for measuring the effects of MTS modification on APP^+^ uptake by SERT or ASP^+^ uptake by DAT and NET using fluorescence image analysis in single live cells.

Strikingly, ASP^+^ has been reported to accumulate in the cells expressing SERT [38], DAT [57], or NET [54] by two phases. The initial phase was characterized by a rapid increase in ASP^+^ fluorescence intensity that was restricted to the plasma membrane, indicating ASP^+^ binding to the cell surface transporters. In the second phase, ASP^+^ fluorescence intensity was intracellularly increased with a slower rate, representing ASP^+^ transport into the cells. A real time and spatial resolved confocal imaging analysis has been used for distinguishing between ASP^+^ uptake and binding by SERT [38].

To determine conformational changes in the cytoplasmic pathway with this method, it is necessary to use an appropriate detergent that can permeabilize the cell membrane to effectively increase accessibility of MTS reagents to the cysteine residues in the cytoplasmic face but keep the ability of transporters to bind its fluorescent ligands. Digitonin, a non-ionic detergent, has been used for solubilization of plasma membrane SERT and DAT without loss of its ligand binding activity [59,60,61]. In addition, it has been reported that digitonin treatment increased accessibility of MTSET and a biotinylating reagent, NHS-SS-biotin, to SERT cysteine or lysine residues at the cytoplasmic face in permeabilized cells [42]. In this study, our results showed that one cysteine residue at the cytoplasmic face, Cys277, is more accessible to MTSEA than MTSET, a reagent with a larger size, in digitonin-permeabilized cells, consistent with a previous observation [60]. Thus, taken advantage of ASP^+^ binding ability with micromolar affinity to SERT, DAT, or NET [38,54,57], fluorescence image analysis can be applied for measurement of conformational changes in the cytoplasmic pathway by MTSEA modification of the cytoplasm-facing cysteine residues in the digitonin-permeabilized cells.

We investigated the role of Cl^−^ ion in the conformational mechanism of SERT by using the cell-based method. Our fluorometric analysis indicates that Cl^−^ acts to decrease accessibility in the extracellular pathway and increase it in the cytoplasmic pathway and that the action of Cl^−^ relies on the presence of Na^+^, consistent with our recent observation with GlyT1 [37]. Hence, we hypothesize that Cl^−^ works with a similar mechanism in the Cl^−^-required NSS transporters to facilitate the conformational conversion from outward to inward open states. Furthermore, we applied this method for assessing the effects of a novel antidepressant drug, vilazodone, on SERT conformation. The conventional antidepressant drugs, such as fluoxetine and paroxetine, are thought to bind to the central binding site, whereas vilazodone has been recently demonstrated to bind to an allosteric site in SERT [23,47,48]. Therefore, it is of interest to compare the conformational mechanism of their action in order to develop novel antidepressant agents that target the conformation of SERT. Our results indicate although these agents bind to the different sites in SERT, they exert a similar effect on SERT conformation in the cell-based assays, in which both the conventional antidepressant drugs and vilazodone have been shown to stabilize SERT in an outward open and inward-closed conformation.

The conformational mechanism by which monoamine transporters mediate conformational changes in response to ion and substrate binding has not been fully addressed. To understand the conformational mechanism of the transporters, we cannot rely on structural biology alone. The cell-based fluorometric method provides an additional tool to explore the conformational changes in the transport cycle in live or permeabilized cells and it is potentially applicable to any monoamine transporters in the NSS family.

## 4. Materials and Methods

### 4.1. Materials

HeLa (CCL-2) and HEK 293T cells were from American Type Culture Collection. C-terminal FLAG-tagged rat SERT C109A, Y107C/C109A, S404C/C109A, and S277C/X5C expression plasmids in pcDNA3.1 were obtained from Dr. Rudnick laboratory, Yale University School of Medicine. Lenti-EF-1α-BSD vector with a blasticidin resistance and two packaging vectors (psPAX2 and pMD2G) were generous gifts from Dr. G. Wang (Guangzhou University). APP^+^ and ASP^+^ were purchased from Sigma-Aldrich. Fluoxetine, paroxetine, citalopram, imipramine, 5-HT, 5-hydroxytryptophan (5-HTP) and L-tryptophan were purchased from Macklin. LipoJet was obtained from Signagen. MTSET and MTSEA were purchased from Biotium. All other reagents were of analytical grade.

### 4.2. Lentivirus and Stable Cell Line Preparation

SERT Y107C and S404C mutants were constructed in the rat SERT C109A background carrying a Flag tag at the C terminus, whereas S277C mutant was generated in X5C, a construct in which five endogenous accessible cysteine residues were replaced [42]. In addition, Ser277 was replaced with cysteine to allow measurement of cytoplasmic pathway accessibility [8,15]. The cDNA sequences encoding full-length of the cysteine mutants in pcDNA3.1 were amplified by PCR and inserted into the BamH I and Xba I sites of lenti-EF-1α-BSD vector by Exnase II, respectively. All mutations in the lentiviral plasmids (Lenti-EF-1α-C109A-BSD, Lenti-EF-1α-Y107C/C109A-BSD, EF-1α-S404C/C109A-BSD, and Lenti-EF-1α-S277C/X5C-BSD) were confirmed by a full-length DNA sequencing.

The lentivirus for WT or each mutant were prepared using HEK 293T cells, respectively, as described previously [62]. In brief, HEK 293T cells at 70-80% confluence were transfected by a mixture of the lentiviral plasmid and other two packaging vectors, psPAX2 and pMD2G, using LipoJet transfection reagent. Viruses were collected into a 10 mL tube 48 h after transfection, followed by centrifuging to remove cell debris at 500× *g* for 10 min. The supernatant was then filtered through a 0.45 μm filter and stored at −80 °C until further use.

HeLa cells were infected by the lentivirus with polybrene in the complete Dulbecco’s Modified Eagle’s Medium (DMEM) with blasticidin S at a concentration of 12 μg/mL. The medium was replaced every 3 days until colonies of blasticidin S-resistant cells were formed. The cells were maintained in DMEM supplemented with 10% fetal bovine serum, 100 units/mL penicillin, 100 μg/mL streptomycin, and 12 μg/mL blasticidin S at 37 °C in a humidified 5% CO_2_ incubator and then plated in 12-well culture plates. The stable cell lines expressing SERT-WT and mutants were confirmed by APP^+^ or ASP^+^ uptake assay and immunoblot analysis for SERT.

### 4.3. APP^+^ or ASP^+^ Accumulation Measurements

The cells stably expressing SERT-WT or mutants were wet mounted on polylysine-coated glass slides and applied for indicated treatments. In brief, the cells were washed twice with 500 μL KRH buffer containing 20 mM HEPES, pH 7.4, 120 mM NaCl, 1.3 mM KCl, 2.2 mM CaCl_2_, 1.2 mM MgSO_4_, and 0.1% (*w*/*v*) glucose. APP^+^ or ASP^+^ accumulation was measured by adding 500 μL KRH buffer containing 2 μM APP^+^ or 10 μM ASP^+^ and incubating for 5 min at 22 °C. Excess APP^+^ or ASP^+^ was then removed by rapid washing three times with 500 μL KRH buffer. The extent of APP^+^ or ASP^+^ accumulated in the cells was determined with confocal imaging analysis. Nonspecific transport for APP^+^ or ASP^+^ was measured in the presence of 10 μM fluoxetine and was subtracted to give APP^+^ or ASP^+^ accumulation, respectively.

### 4.4. Fluorescence Image Acquisition and Fluorescence Intensity Analysis

Images were acquired at 22 °C using a 20 or 60 × water immersion objective with the Zeiss LSM 900 confocal microscope. APP^+^ and ASP^+^ were excited by an argon laser with excitation peak at 488 nm. APP^+^ fluorescence was captured at 490–565 nm and ASP^+^ fluorescence at 600–650 nm. Images were analyzed using National Institutes of Health ImageJ v1.53q. Fluorescence intensity in each cell was counted and normalized to the cell area (mean fluorescence). To obtain ASP^+^ binding on the cell surface, intracellular ASP^+^ fluorescence intensity was subtracted from total fluorescence in each cell, and the resulting ASP^+^ fluorescence on the cell surface was then normalized to the cell membrane area.

### 4.5. Cystine Accessibility Measurements

Accessibility of the cysteine residues in the extracellular pathway was measured by reactivity of Y107C/C109A and S404C/C109A mutants with MTSET. The cells stably expressing Y107C/C109A or S404C/C109A were incubated with MTSET over a range of concentrations in HEPES buffer containing indicated ligands or ions at 22 °C for 15 min. After excess MTSET and ligands were removed by rapid 3 × washing with the same buffer, the cells were then incubated with APP^+^ at a final concentration of 2 μM in KRH buffer at 22 °C for another 5 min. The extent of APP^+^ accumulated in the cells was determined by confocal imaging analysis in the live cells. To measure the effects of antidepressants, substrate 5-HT, or other ligands such as 5-HTP or tryptophan, where added, was present at 10 μM. To measure the effects of ions, 150 mM N-methyl-D-glutamine (NMDG) gluconate, and NMDG chloride, sodium isethionate, or NaCl was used as indicated. These measurements of reactivity depend on the ability of MTSET to inactivate APP^+^ transport activity by an allosteric mechanism [41]. The MTSET concentration causing half-maximal inactivation was determined and used to calculate the rate constant for cysteine modification, as described previously [8,16].

For cytoplasmic pathway accessibility, measurements were performed with the cells stably expressing S277C/X5C. The cells were treated with MTSEA at a range of concentrations in HEPES buffer containing indicated ligands or ions in the presence of 25 μg/mL digitonin at 22 °C for 5 min. The cells, then, were washed free of unreacted MTSEA and ligands, ASP^+^ binding was measured by adding 500 μL KRH buffer containing 10 μM ASP^+^ and incubating at 22 °C for 5 min. Excess ASP^+^ was removed by 3 × rapid washing with KRH buffer and ASP^+^ fluorescence retained in the cell membrane was measured by confocal imaging analysis in the permeabilized cells. Non-specific ASP^+^ binding was measured by adding 10 μM fluoxetine. The MTSEA IC_50_ value was determined and used to calculate the rate constant for S277C/X5C modification.

### 4.6. Data Analysis

Nonlinear regression fits of experimental and calculated data were performed with Origin (Origin Lab). The statistical analysis given was from multiple experiments. Data with error bars in the figures represent the mean ± SD for at least 10 measurements per condition in one experiment or the mean ± SEM for three experiments as indicated, respectively. Statistical analysis was performed using Student’s paired *t* tests.

## 5. Conclusions

The present work developed a new method for determining conformational changes in SERT with its fluorescent substrates by using fluorescence image analysis in live or permeabilized cells. We validated this method by investigating ion, substrate, and other ligand-induced conformational changes in both the extracellular and cytoplasmic pathways of SERT. Consistent with the previous experimental results obtained from radioactive assays with intact cells and cell membranes, the cell-based fluorometric analysis verified the conformational mechanism for alternating access in SERT. Furthermore, we applied this method for examining the role of Cl^−^ ion in the conformational mechanism and the effects of vilazodone, a novel antidepressant drug, on SERT conformation. Our results showed that Cl^−^ ion, in the presence of Na^+^, facilitates the conformational conversion from outward to inward open states, and that vilazodone binding to the allosteric site stabilizes SERT in an outward open and inward-closed conformation. The present work provided insights into the conformational mechanism of SERT and also indicated that the cell-based fluorometric method is robust, straightforward to perform, and potentially applicable to any monoamine transporters in exploring the transport mechanism and mechanism of action of therapeutic agents for the treatment of several psychiatric disorders.

## Figures and Tables

**Table 1:** Kinetic parameters of WT-SERT and cysteine mutants.

	APP^+^		ASP^+^	
	*K_m_* (μM)	*V_max_* (AFU)	*K_m_* (μM)	*V_max_* (AFU)
WT	2.63 ± 0.06	30.5 ± 1.4	8.52 ± 0.15	75.8 ± 5.3
Y107C/C109A	2.39 ± 0.04	39.4 ± 1.8	9.32 ± 0.48	59.9 ± 4.2
S404C/C109A	2.07 ± 0.04	34.9 ± 1.2	8.87 ± 0.59	53.2 ± 3.8 *
S277C/X5C	1.99 ± 0.05	26.5 ± 1.1	9.66 ± 0.94	40.2 ± 3.0 **

Kinetic analysis for APP^+^ or ASP^+^ uptake was performed by incubating the cells stably expressing WT-SERT or each mutant with APP^+^ or ASP^+^ over a range of concentrations at 22 °C for 5 min. After washing to remove excess APP^+^ or ASP^+^, the accumulated fluorescence within the cells (*n* ≥ 30) were counted and normalized to the cell areas, as described under “Materials and Methods”, respectively. These calculated *K_m_* and *V_max_* values represent mean ± SEM of three experiments. Asterisks indicate statistically significant changes compared to the WT. * *p* < 0.05; ** *p* < 0.01.

## Data Availability

The data presented in this study are available on request from the corresponding author.

## Supplementary Materials

The supporting information can be downloaded at: https://www.mdpi.com/article/10.3390/ijms231810919/s1.

## Abbreviations

SERT: serotonin transporter; 5-HT, 5-hydroxytrypatamine; NSS, neurotransmitter sodium symporters; APP^+^, 4-[4-(dimethylamino) phenyl]-1-methylpyridinium; ASP^+^, 4-(4-(dimethylamino) styryl)-N-methylpyridinium; MTSET, 2-(Trimethylammonium) ethyl methanethiosulfonate bromide; MTSEA, 2-aminoethyl methanethiosulfonate hydrobromide; LeuT, leucine transporter; NMDG, N-methyl-D-glutamine.
