# Supplementary material for: Determining Ligand and Ion-Induced Conformational Changes in Serotonin Transporter with Its Fluorescent Substrates
Source: Int J Mol Sci. 2022 Sep 18;23(18):10919. doi: 10.3390/ijms231810919 (PMC9503009; doi:10.3390/ijms231810919)
Supplement: Supplementary file 1 [file ijms-23-10919-s001.zip › ijms-1913571-supplementary.pdf]

Supplemental Materials

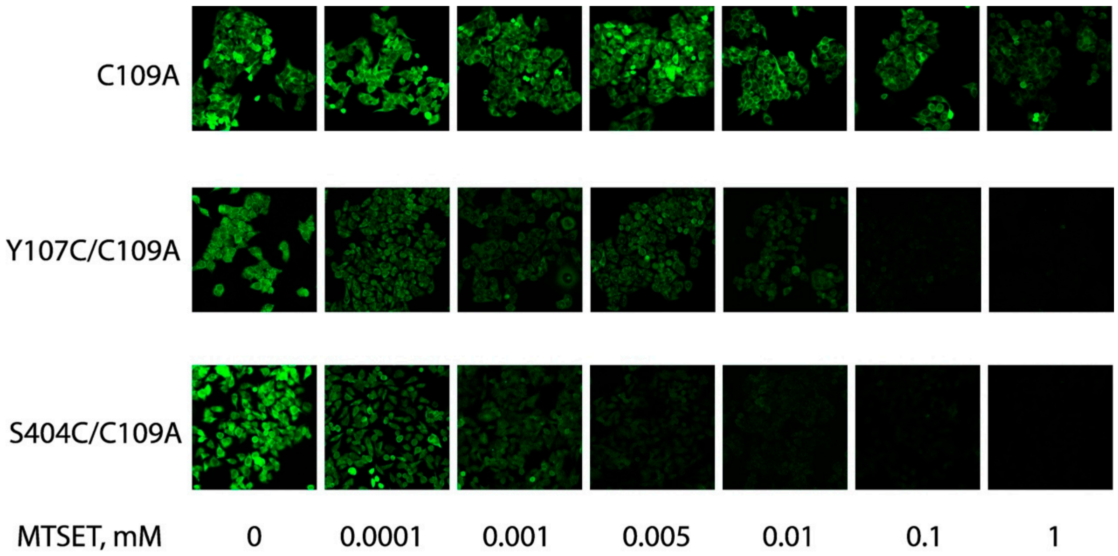

Figure S1. Representative confocal fluorescence images of APP^+ uptake by C109A, Y107C/C109A, or S404C/C109A under various MTSET concentrations. The experiment was repeated twice with similar results.

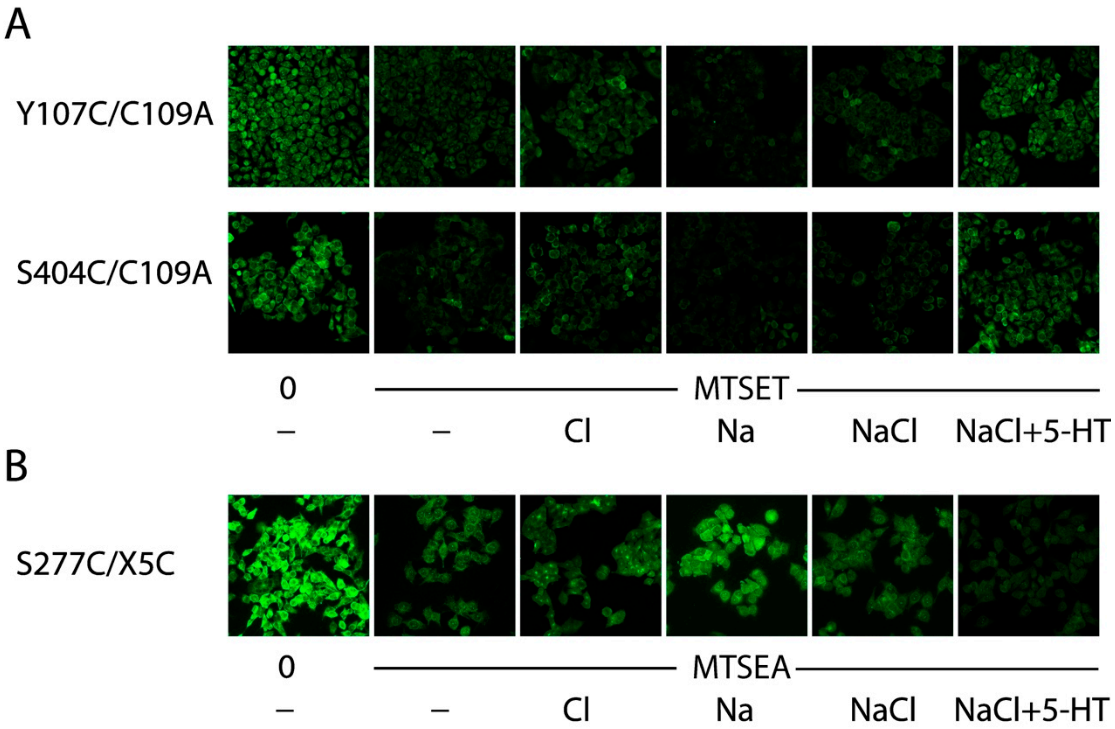

Figure S2. Representative confocal fluorescence images of APP^+ uptake by Y107C/C109A and S404C/C109A **(A)** or ASP^+ binding by S277C/X5C **(B)** under various ion conditions. The experiment was repeated twice with similar results.

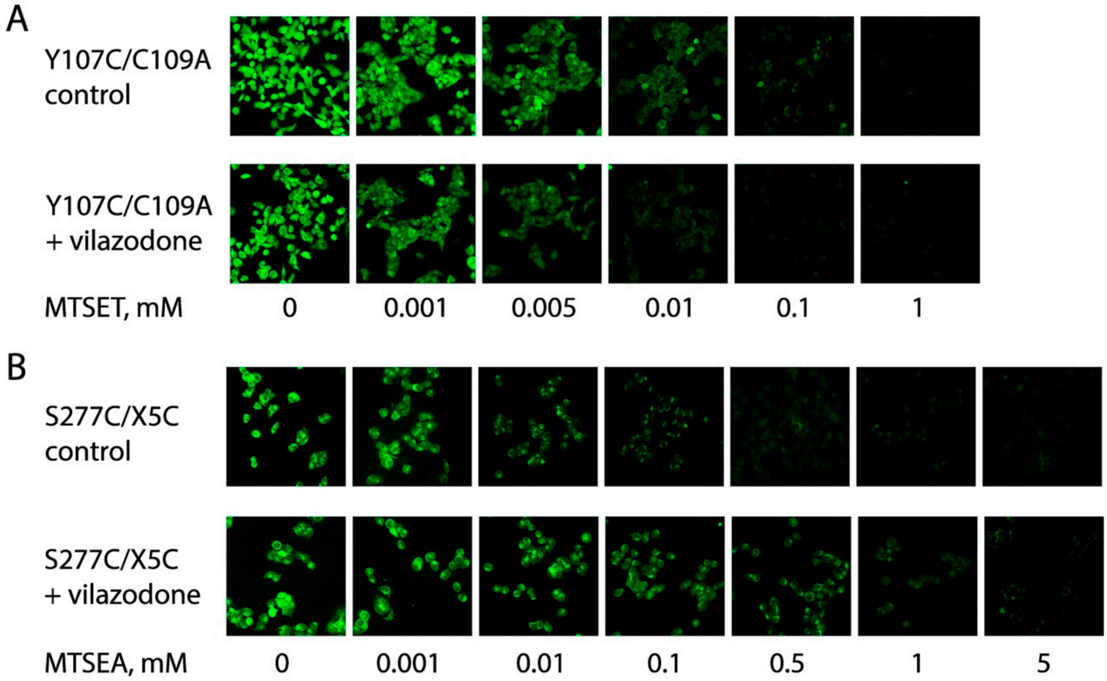

Figure S3. Representative confocal fluorescence images of APP^+ uptake by Y107C/C109A **(A)** and ASP^+ binding by S277C/X5C **(B)** under various MTSET or MTSEA concentrations in the absence or presence of 10 μM vilazodone. The experiment was repeated twice with similar results.
